# Sexual Dysfunction and Quality of Life in Chronic Heroin-Dependent Individuals on Methadone Maintenance Treatment

**DOI:** 10.3390/jcm8030321

**Published:** 2019-03-07

**Authors:** Carlos Llanes, Ana I. Álvarez, M. Teresa Pastor, M. Ángeles Garzón, Nerea González-García, Ángel L. Montejo

**Affiliations:** 1 Department of Psychiatry, Complejo Asistencial de Zamora, Zamora 49022, Spain; 2 Department of Psychiatry, Hospital Clínico Universitario de Salamanca, Salamanca 37007, Spain; aialvarez@saludcastillayleon.es (A.T.Á.); magarzon@saludcastillayleon.es (M.Á.G.); 3 Castilla y León Health Authority, Complejo Asistencial de Zamora, Zamora 49022, Spain; mtpastor@saludcastillayleon.es; 4 Department of Statistics, University of Salamanca, Institute of Biomedical Research of Salamanca IBSAL, Salamanca 37007, Spain; nerea_gonzalez_garcia@usal.es; 5 Psychiatry, University of Salamanca, Institute of Biomedical Research of Salamanca IBSAL, Salamanca 37007, Spain; amontejo@usal.es

**Keywords:** opioid-related disorders, methadone, adverse effects, erectile dysfunction, medication adherence

## Abstract

This study examined whether methadone (hereinafter referred to as MTD) maintenance treatment (MMT) is correlated with sexual dysfunction (SD) in heroin-dependent men. This was conducted to determine the prevalence of sexual dysfunction and if there is a relationship between duration and dose among men on MMT and its impact on the quality of life. The study combined a retrospective and a cross-sectional survey based on the Kinsey Scale, TECVASP, and PRSexDQ-SALSEX clinical interviews of 85 patients who are currently engaged in MMT. Sexual dysfunction in all five PRSexDQ-SALSEX domains (lack of libido, delay in orgasm, inability to orgasm, erectile dysfunction, and tolerance or acceptance of changes in sexual function) was associated with dose and long-term use of heroin. All dimensions of SD were affected by the MTD intake. From the analysis of our sample, we may conclude that dose of MTD and overall score of SD were directly associated. However, no evidence was found to prove that treatment duration and severity of SD were linked. It is notable that only one tenth of the patients spontaneously reported their symptoms of the sexual sphere, but up to a third considered leaving the MMT for this reason.

## 1. Introduction

Opioid dependence is a rising drug use disorder with a substantial contribution to the global disease burden. The absolute number (age standardized prevalence) of people with opioid dependence worldwide increased from 10.4 million (0.20%) in 1990 to 15.5 million (0.22%) in 2010, and the disability adjusted years of life lost attributable to opioid dependence rose from 5.3 million (0.21% of global disease burden) in 1990 to 9.2 million (0.37%) in 2010 [1]. Opioid substitution treatment, either with methadone or buprenorphine, has been shown to be safe and effective in suppressing illicit opioid use, improving physical and mental wellbeing, and reducing all cause and overdose mortality [2]. However, methadone is more commonly used for maintenance treatment [3]. Methadone maintenance treatment (MMT) is a comprehensive treatment program that involves the long-term prescribing of methadone as a substitution therapy for opioid dependence. Despite the effectiveness of the methadone maintenance treatment [4], previous studies have found that sexual dysfunction, including hypoactive sexual desire disorder, erectile dysfunction, and orgasmic dysfunction, is common in heroin users and individuals being treated for heroin addiction [5]. In a recent meta-analysis, the meta-analytical pooled prevalence for sexual dysfunction among methadone users was 52% (95% confidence interval, 0.39–0.65). Hypoactive sexual desire disorder and low libido were the most prevalent sexual dysfunctions, accounting for 51% of cases [6]. Several hypotheses have been suggested to explain the correlation between methadone use and sexual dysfunction. One well-known hypothesis is that methadone exerts neuroendocrinological effects on the tubero-infundibular and hypothalamic-pituitary-gonadal axes. The chronic stimulation of the μ-opioid receptors by methadone alters the function of the tubero-infundibular axis and the dopaminergic control of prolactin, with a consequential impact on sexual functioning [7]. A high level of circulating prolactin causes the inhibition of the gonadotropin-releasing hormone, which lowers the levels of sex hormones, especially testosterone. Men with low testosterone levels may exhibit a decrease in sexual interest [8]. A recent qualitative study has found that some MMT subjects who experienced sexual dysfunction chose to withdraw from interactions with their partners, which led to conflicts. Such conflicts negatively impacted the rehabilitation. Furthermore, inappropriate reactions to the sexual problems included premature treatment discontinuation under pressure from partners, methadone dose reduction, and the use of other illicit drugs to enhance sexual performance [9]. The measurement of the health-related quality of life construct (HRQoL) is widely used in the field of health. This represents individual responses to the physical, mental, and social effects that a health alteration produces on daily life. In drug addiction, this construct has been used for a relatively short time [10]. 

Although sexual dysfunction is not life threatening, it may often result in withdrawal from sexual intimacy, thereby reducing quality of life [11]. Therefore, we conducted this study to investigate sexual dysfunction in men and women on MMT. We also investigated the correlation and association between sexual dysfunction and quality of life in this group of patients.

## 2. Experimental Section

Methods and study design: this cross-sectional study was conducted in the drug detoxification unit in Complejo Asistencial de Salamanca Hospital in Salamanca, Spain, which is the Castilla y León regional reference unit for the treatment of addictions in hospitalization. The research period was from May 2017 to October 2018. 

Participants: all participants were recruited on admission to the drug detoxification unit to voluntarily withdraw or reduce methadone. Subjects were eligible for this study if: (1) They were men or women over 18 years old; (2) they had been engaged in MMT; (3) they had a diagnosis of mental and behavioral disorders due to the use of opioids (F11.3 ICD-10); (4) their urine was found to be negative in drug use in the weekly analytical control for the six months prior to admission; and (5) they were not under treatment with any psychodrugs except benzodiazepines.

Interviews and measures: an original form was developed to record the information of the participants. The questionnaire included items on demographic characteristics (sex and age) and methadone treatment status (such as the time of receiving MMT and methadone dose). It also include if they were being treated with benzodiazepines or not and if they were, the equivalent dose in diazepam.

The Kinsey scale [12], also called the Heterosexual–Homosexual Rating Scale, is used to describe a person’s sexual orientation based on their experience or response at a given time. It consists of nine items that explore the importance of sexual life for the patient and the degree of satisfaction with it, the identification of the patient with the different groups of sexual orientations, and the frequency of sexual intercourse of the patient. The first seven items are answered on a scale of 1 to 5 and the last two are of a dichotomous nature. The scale is self-applied and typically ranges from “0”, Exclusively heterosexual to “1”, Predominantly heterosexual, only incidentally homosexual; “2”, Predominantly heterosexual, but more than incidentally homosexual; “3”, Equally heterosexual and homosexual; “4”, Predominantly homosexual, but more than incidentally heterosexual; “5”, Predominantly homosexual, only incidentally heterosexual; and “6”, Exclusively homosexual. In both the male and female volumes of the Kinsey Reports, an additional grade, listed as “X”, indicated no socio-sexual contacts or reactions.

PRSexDQ-SALSEX is a brief and clinician-administered questionnaire that includes seven questions in total [12]. The presence of sexual function impairment in patients with psychiatric disorders is very common and could be an effect of the medication (mainly antidepressants and neuroleptics) [13]. Questions A and B are screening items used to assess whether the patient had noticed changes in sexual function since pharmacotherapy or during the last four weeks and reported it spontaneously. Items 3–7 are questions evaluating five dimensions of SD on a scale of 0–3: loss of libido, delayed orgasm or ejaculation, lack of orgasm or ejaculation, erectile dysfunction in men/vaginal lubrication dysfunction in women, and patient’s tolerance. The total score of PRSexDQ-SALSEX ranges from 0 to 15 [14]. 

For the measurement of the health-related quality of life (HRQoL), the test specifically designed for the drug-dependent population TECVASP [15] was used. TECVASP (acronym in Spanish of Test for the Evaluation of the Quality of Life in Addicts to Psychoactive Substances) consists of 22 items (18 positive and four negative [items 15, 19, 20, and 21]), with a graduated response format of five alternatives. The response alternatives are coded with the following scores: (a) in the positive items: nothing (5 points), little (4 points), sometimes (3 points), enough (2 points), and a lot (1 point); (b) in the negative items: nothing (1 point), little (2 points), sometimes (3 points), enough (4 points), and a lot (5 points). In this way, for each item, a higher score represents a more positive assessment of the content, and in the test, a higher score represents a better HRQoL.

Statistical Analysis: Kolmogorov-Smirnov was one sample test used to examine the quantitative variables’ distribution. Normally distributed variables were described as mean ± standard deviation; otherwise, their information was summarized by median ± interquartile range. Categorical response features were measured through absolute or relative frequencies and percentages. Differences between two independent groups were tested by the Student’s *t*-test (for normal distribution data) or Mann-Whitney *U* test. Comparisons of more than two independent groups were studied by ANOVA (for data with parametric distribution) or the Kruskal-Wallis test (for non-normal distributions). The Chi-Square test, Fisher’s exact test, and tau de Kendall measure were used to evaluate the association in qualitative variables. 

Patients were classified based on the categorization of SALSEX total score into four different groups: no Sexual Dysfunction (SD) (a score of 0 points), mild SD (a score of 1–5 points, where no items scored ≥ 2), moderate SD (a score of 6–10 points or an item scoring 2 and no items scoring 3), or severe SD (a score of 11–15, or any items scoring 3 points). In addition, based on the cut-off points of the first and third quartiles, the dose of methadone (MTD) consumed was categorized as <30 mg, 30–60 mg, and >60 mg. Subsequently, Correspondence Factor Analysis (CFA) was used to analyze the relationship between methadone consumption and sexual problems, taking into account tolerance, dose, and time of methadone treatment. CFA is a statistical technique that produces a graphical representation of a contingency table, facilitating the interpretation of the association between two categorical variables. The categories of these variables are represented by points on a plane. For a correct interpretation of a CFA representation, it should be taken into account that two close points in the graph refer to positive associated categories. 

Finally, relationships between sexual activity and quality of life of patients based on SD severity groups were studied. Differences in questions with five Likert response options were evaluated by means of an ANOVA or Kruskal-Wallis test, since this treatment is admitted when the number of Likert alternatives is greater than four. The global quality of life score was computed by the sum of the 22 items’ scores of the TECVASP scale. Here, it is important to note that: (i) there were 18 inverse items and four direct questions; (ii) scores’ range vary from 22 to 110 points; and (iii) the higher the score in the test, the worse the quality of life of patients.

## 3. Results

### 3.1. Clinical and Sociodemographic Characteristics of Patients

Patients’ characteristics are shown in Table 1. The sample consisted of 85 patients, mainly men (*n* = 72, 84.7%), with a mean age of 43.1 ± 7.7 years, with 23 years being the youngest patient and 58 years the oldest. The mean dose of methadone consumed by patients was 49.01 ± 29.87 mg, with a mean treatment time of 7.21 ± 6.95 years. Differentiating by sexes, the mean age of men was 42.76 ± 7.79 years, and the mean duration of treatment and mean dose were 6.34 ± 6.74 years and 45.33 ± 25.29 mg, respectively. For women, the mean age was 44.85 ± 7.2 and they consumed a mean dose of 69.38 ± 43.92 mg, during 9.85 ± 7.78 years of treatment.

About half of the patients from the sample suffered from a personality disorder (*n* = 49, 57.6%). One-third of the patients consumed self-administered benzodiazepines (31.8%; mean dose 95.2 ± 98.5 mg), while one quarter took benzodiazepines by medical prescription (25.9%; mean dose 36.4 ± 32.7 mg). There was a significant statistical difference between the doses of self-administered and medicated doses of benzodiacepines (*p* = 0.001), with the self-administered benzodiazepines being the greater dose. None of the patients taking self-administered benzodiazepines had received medical advice to get them.

#### Differences between Sexes in Clinical and Sociodemographic Characteristics

Table 2 contains features of patients according to their sex. During their treatment time, which was significantly different in men and women (*p* = 0.01), men received a lower dose of MTD. Tolerance toward MTD was significantly better in men than women, who presented low percentages of good tolerance. Although the doses of both self-administered and medically prescribed benzodiazepines were lower in women, no statistically significant differences were observed by sex. No age difference was found between sexes.

### 3.2. Sexual Activity, Frequency of SD and Group Differences in SALSEX Scores

83.5% of the participants described themselves as heterosexual, 35.3% had an exclusively monogamous sexual relationship, and 23.5% had non-monogamous sexual relations. Furthermore, 15.3% had sexual intercourse in the last semester to get money or cover a material need, or paid for sexual intercourse (24.7%). Kinsey results showed that a high percentage of the sample gave importance to sex (71.7%), but only 24.7% were satisfied with their sexual activity.

**Figure A1:**
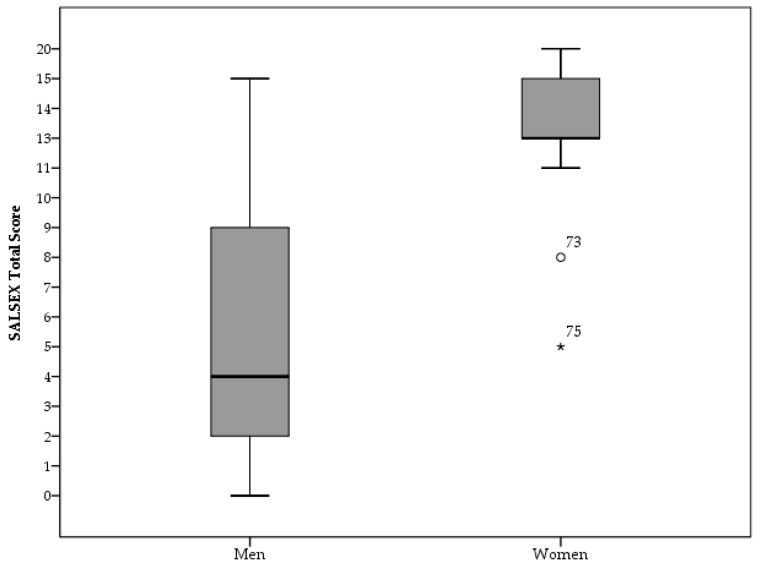
Difference between men and women in SALSEX total score. The ° symbol represents an outlier and the * symbol corresponds to extreme value.

At the same time, a total of 85.9% of the patients in this investigation suffered from SD. Among them, 24.7% showed mild SD, 21.2% suffered from moderate SD, and 40% had severe SD. There was a highly significant difference between men and women in terms of the PRSexDQ-SALSEX total score (*p* = 0.000; Figure A1), being more serious in female patients. 76.5% felt alteration in their sexual activity after the beginning of methadone treatment, but only 11.8% reported it to the doctor without being questioned. In addition, 76.9% of patients were disturbed by SD and 32.7% of them considered interrupting the MTD treatment.

**Figure 1:**
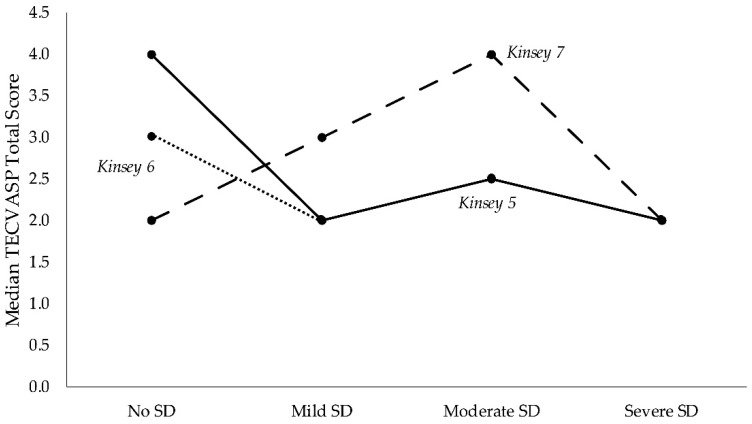
Differences in Kinsey questionnaire items, distinguished by the four SD classes of SALSEX.

Dysfunction groups showed a statistically different behaviour in items 5 (*p* = 0.037), 6 (*p* = 0.003), and 7 (*p* = 0.005) of the Modified Kinsey scale. Patients without sexual dysfunction had sex more frequently than patients with SD (Figure 1, median values of items 5 and 6), with the patients having severe sexual dysfunction having the lowest score.

#### Impact of Treatment Duration, Methadone Dose Consumed, Tolerance toward Methadone, and Effect of Using Benzodiazepines on Sexual Dysfunction

Generally, correlation analysis allowed us to conclude that the dose of MTD and overall score of SD were directly associated (Pearson correlation coefficient 0.332; *p* = 0.002). However, no evidence was found that treatment duration and severity of SD were linked (Pearson correlation coefficient 0.183; *p* = 0.094).

The frequency of SD problems by dose and tolerance toward MTD are summarized in Table 3 and Table 4. Differences (Kruskal-Wallis analysis) between the four SD groups of patients (no SD, mild SD, moderate SD, severe SD) showed statistically significant dissimilarities on MTD’s dose (*p* = 0.005) and MTD’s tolerance (*p* = 0.000). Patients with severe sexual dysfunction were those who took higher doses of MTD, as well as those who showed the worst tolerance towards the opiate. All cases with poor tolerance toward MTD presented severe sexual dysfunction.

In Table 4, the percentage of patients suffering from some libido, eyaculation/orgasm, anorgasmia, or erection/lubrication problem are summarized, regardless of whether the symptoms were mild. Patients who did not suffer from SD, did not present problems in any of the evaluated dimensions (decreased libido (DL), delay in eyaculation/orgasm (DEO), anorgasmia (A), and erection/lubrication problem (ELP)). However, all dimensions of SD were affected by the MTD intake in those with mild, moderate, or severe SD. Patients treated with a lower amount of MTD suffered from less problems of delay in ejaculation/orgasm or in the inability to ejaculate/have orgasm during intercourse. The same occurred with those patients with a better tolerance to this opiate. The dose associated with the highest erection/lubrication problem (94.1%) was more than 60 mg of MTD.

**Figure 2:**
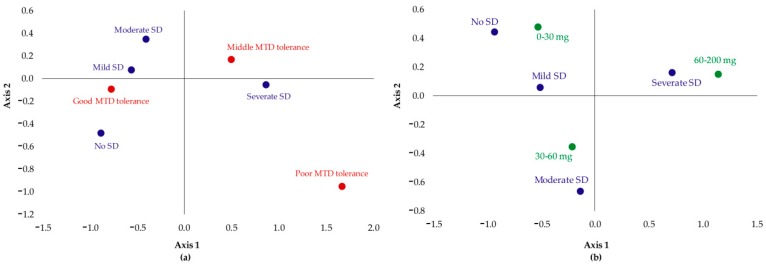
CFA graphical representations of the categories of two qualitative variables: (**a**) Tolerance to MTD (red) vs severity of SD (blue); (**b**) dose of MTD (green) vs severity of SD (blue).

As seen in the CFA graphical representation (Figure 2a) and remembering that the proximity between points can be understood as a direct association between categories, it was observed that:

- Patients which had no SD or their SD was not severe showed good tolerance to MTD;
- Patients with moderate SD were those that had good or medium tolerance;
- Severe SD was associated with poor MTD tolerance.

After examining the influence of mg of dose with the grade of dysfunction (Figure 2b), it could be concluded that:

- Patients which had no SD or their SD was mild were those who took a dose between 0 and 30 mg;
- Patients with moderate SD took 30–60 mg of MTD;
- Severe SD was associated with the highest doses: 60–200 mg.

**Figure A2:**
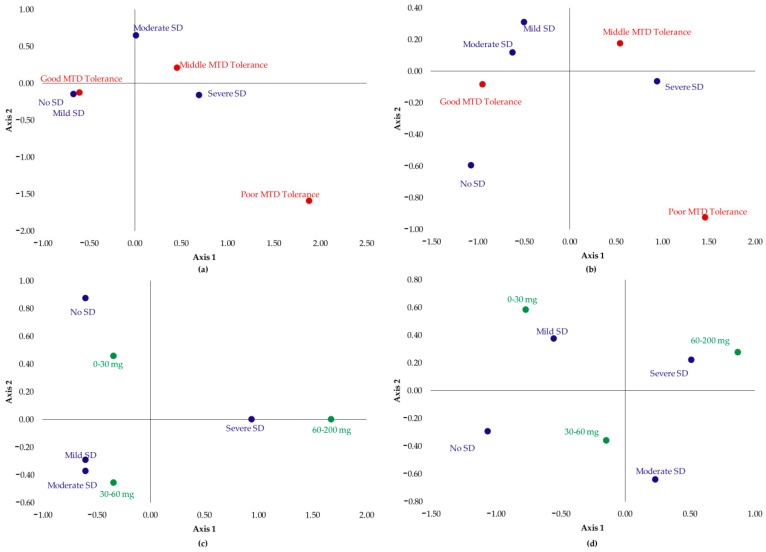
CFA graphical representations of the categories of two qualitative variables: (**a**) Tolerance to MTD (red) vs severity of SD (blue) of patients that only use MTD; (**b**) tolerance to MTD (red) vs severity of SD (blue) of patients that use MTD and benzodiazepines; (**c**) dose of MTD (green) vs severity of SD (blue) of patients that only use MTD; (**d**) dose of MTD (green) vs severity of SD (blue) that use MTD and benzodiazepines.

Finally, 57.6% of the patients also used benzodiazepines (both self-administered and medically prescribed). Due to this last characteristic, the presence of DS was then examined depending on whether the patients used methadone alone or methadone combined with benzodiazepines. Firstly, SALSEX scores difference analysis between both groups presented non-significant differences (*p* = 0.242). In other words, no significant evidence was found to corroborate that the use of benzodiazepines combined with methadone influences the presence and/or severity of DS. Secondly, CFA graphical representation (Figure A2) shows the differences in the association between DS and doses of MTD and DS and tolerance to MTD in patients who only consume MTD (panels a and c) and patients who consume both methadone and benzodiazepines (panels b and d). In the case of patients who only take MTD the association between not suffering from SD or mild SD and having a good tolerance to MTD is very strong (panel a), while those who consume MTD and benzodiazepines suffer from moderate SD, although they have a good tolerance to MTD (panel b). Regarding the dose consumed, patients taking a dose of 0–30 mg of MTD and also benzodiazepines (panel d) are associated with mild SD, while patients who only take MTD (dose of 0–30 mg) are associated with a diagnosis of no presence of DS (panel c).

### 3.3. Quality of Life in Presence of Sexual Dysfunction Problems

The mean score of the quality of life test TECVASP was 58.12 ± 13.74 points. Statistically significant differences were found for SALSEX classes in questions 4 (*p* = 0.006), 10 (*p* = 0.019), 14 (*p* = 0.034), 16 (*p* = 0.019), and 22 (*p* = 0.005). 

**Figure A3:**
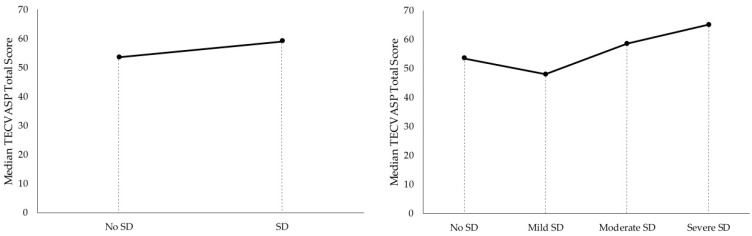
Quality of life based on SALSEX classes (no SD, mild SD, moderate SD, and severe SD).

Figure A3 contains the global quality of life score test for patients that had no SD versus patients which suffered from SD of any grade. The median score of the SD group was higher than the median score of patients without dysfunction problems, mirroring the results of worse quality of life in those patients. A comparison of four classes of SD reported a significant difference (*p* = 0.011, Figure A2b), where patients with severe sexual dysfunction suffered from the worst quality of life and patients with mild sexual dysfunction had the best quality of life.

## 4. Discussion

The Kinsey scale results showed that a high percentage of the sample gave importance to sex (71.7%), but only 24.7% were satisfied with their sexual activity. Patients in MMT had problems with sexual function in one or more of the five PRSexDQ-SALSEX domains (loss of libido, delayed orgasm or ejaculation, lack of orgasm or ejaculation, erectile dysfunction in men/vaginal lubrication dysfunction in women, and patient’s tolerance.). The research literature has noted high rates of sexual dysfunction in heroin users and MMT patient populations [14]. Our study found that 85.9% of participants in MMT had sexual dysfunction (40% of them severe dysfunction), which is perhaps higher than reported in other studies [15,16], although the results found varied greatly [17,18].

Older age, for example, was highly hypothesized to be correlated with increased sexual dysfunction in patients receiving methadone [19]. In our sample, we did not find statistically significant differences between the presence of sexual dysfunction and age (*p* = 0.871). There are also significant differences (*p* = 0.005) in the methadone dose among patients who presented mild, moderate, or severe sexual dysfunction. This finding agrees with what was found by others [11], who showed that patients on higher methadone doses had more sexual dysfunction, and differed slightly from others [14].

Although it is commonly accepted that sexual dysfunction is a direct pharmacological effect of opioids, recent studies have revealed that the etiology of sexual dysfunction in methadone-maintained patients is rather complex [20,21]. Some research has suggested that heroin, amphetamine, alcohol, tobacco, and marijuana can cause sexual dysfunction by a number of mechanisms, including effects on the male reproductive system at the level of the hypothalamus, the pituitary gland, and the testes [22,23]. Additionally, because of this, those patients who had positive toxic control in the six months prior to our study were excluded from the sample. Other factors commonly pointed out are psychological factors (i.e., psychiatric symptoms). To avoid this, we have excluded all those patients who had a psychiatric diagnosis, apart from a substance use disorder (except for personality disorder). The diagnosis of personality disorder appears in 57.6% of the sample, which in itself is linked to sexual dysfunction [24,25], however, and as a limitation of this research, we did not consider physical health and biological factors (i.e., sex hormone), which also significantly contribute to it [26].

There were several limitations to our study. Firstly, we found that a proportion of the participants in MMT used benzodiazepines (both self-administered and medically prescribed) during methadone treatment. This was the only admitted psychopharmacological treatment, and although its presence may interfere with the results [27] (benzodiazepines are psychotropic drugs with some adverse sexual effects) [28], it is not easy to find patients on methadone treatment in monotherapy. Secondly, another limitation of the study is bias due to its retrospective design (instead of prospective one); however, the relatively high mean duration of methadone treatment (6.34 ± 6.74 and 9.85 ± 7.78 years of treatment for men and women, respectively) avoids bias in the recalling symptoms of sexual dysfunction prior to initiating MMT, which would influence the result of this study. We emphasize that they did not take antidepressants or antipsychotics, which have been shown in the literature to cause many sexual dysfunctions [29].

Sexual functioning is critical for improving the quality of life in patients enrolled in an opioid rehabilitation program. The methadone treatment programs should be progressively oriented to the person, valuing their opinions, encouraging their active participation in the process, and improving their quality of life levels, so that the approach to their problems is similar to that of any another health issue.

Clinical implications: clinicians may consider asking about sexual dysfunction while treating heroin dependents, as only 11.8% of the patients in our sample reported it without being questioned about it; however, 32.7% of them considered interrupting the treatment for this reason. As the results of our study showed high rates of sexual dysfunction secondary to methadone treatment (not spontaneously reported), we think it is very important to use scales like these, including brief and relatively nonintrusive questionnaires that ease the exploration and detection of these symptoms and avoid discontinuation of the treatment

## 5. Conclusions

A high prevalence of sexual life dissatisfaction was found in our sample. It suggests that the sexual dysfunction of MMT patients deserves special attention from specialists of addiction treatment settings.

## Figures and Tables

**Table 1:** Clinical and sociodemographic characteristics.

Characteristic	*N* = 85
Age (years)	43.1 ± 7.7
<30 years, *n* (%)	6 (7.1)
30–40, *n* (%)	22 (25.9)
40–50, *n* (%)	45 (52.9)
>50, *n* (%)	12 (14.1)
Sex (males), *n* (%)	72 (84.7)
Treatment time (years)	7.21 ± 6.95
MTD ^1^ dose	49.01 ± 29.87
MTD ^1^ tolerance	
Good tolerance, *n* (%)	37 (43.5)
Middle tolerance, *n* (%)	44 (51.8)
Poor tolerance, *n* (%)	4 (4.7)
Personality disorder, *n* (%)	49 (57.6)
Consumption of self-administered benzodiazepines, *n* (%)	27 (31.8)
Dose (mg)	95.19 ± 98.45
Consumption of medicated benzodiazepines, *n* (%)	22 (25.9%)
Dose (mg)	36.36 ± 32.74

^1^ MTD, Methadone.

**Table 2:** Comparison of sexes in clinical and sociodemographic characteristics.

Characteristic	Men (*n* = 72)	Women (*n* = 13)	*p*-Value
Age (years)	42.76 ± 7.79	46 ± 15.5	0.49
Treatment time (years)	6.74 ± 6.74	9 ± 17.5	0.01
MTD’ dose (mg)	45.33 ± 25.29	60 ± 23.5	0.20
MTD’s tolerance	-	-	0.01
Good tolerance, *n* (%)	35 (48.6)	2 (15.4)	
Middle tolerance, *n* (%)	35 (48.6)	9 (69.2)	
Poor tolerance, *n* (%)	2 (2.8)	2 (15.4)	
Personality disorder, *n* (%)	41 (56.9)	8 (61.5)	0.77
Dose of self-administered benzodiazepines (mg)	96.46 ± 103.78	85 ± 44.44	0.74
Dose of medicated benzodiazepines (mg)	40 ± 36.52	20 ± 40.0	0.59

**Table 3:** Comparison of clinical characteristics between patients with no SD and patients with SD, distinguishing those with mild, moderate, and severe SD.

Characteristic	No SD Patients (*n* = 12)	SD Patients (*n* = 73)			*p*-Value
		Mild (*n* = 21)	Moderate (*n* = 18)	Severe (*n* = 34)	
Age (years)	44 ± 9.75	43 ± 11	45.5 ± 10	42.5 ± 10.5	0.871
MTD’s dose (mg)	32.5 ± 13.75	40 ± 28	39 ± 18.75	53 ± 40.75	0.005
MTD’s tolerance					
Good tolerance, *n* (%)	9 (75)	13 (61.9)	10 (55.6)	5 (14.7)	0.000
Medium tolerance, *n* (%)	3 (25)	8 (38.1)	8 (44.4)	25 (73.5)	
Poor tolerance, *n* (%)	-	-	-	4 (11.8)	
Personality disorder, *n* (%)	8 (66.7)	11 (52.4)	10 (55.6)	20 (58.8)	0.947

**Table 4:** Effect of MTD dose and tolerance on the frequency (%) of sexual dysfunction problems.

Characteristic	No SD Patients (*n* = 12)	Mild SD Patients (*n* = 21)				Moderate/Severe SD Patients (*n* = 52)			
		DL	DEO	A	ELP	DL	DEO	A	ELP
MTD dose									
<30 mg	0	87.5	75	12.5	50	100	72.7	54.5	81.8
30–60 mg	0	72.7	72.7	18.2	27.3	95.8	79.2	83.3	83.3
>60 mg	-	0	100	50	0	94.1	94.1	88.2	94.1
MTD tolerance									
Poor	-	-	-	-	-	100	100	100	100
Medium	0	62.5	75	0	12.5	93.9	87.9	87.9	87.9
Good	0	76.9	76.9	30.8	46.2	93.3	66.6	53.3	80

Decreased libido (DL); Delay in ejaculation/orgasm (DEO); Anorgasmia (A); Erection/lubrication problem (ELP).
